# Fat-suppressed MR Imaging of the Spine for Metal Artifact Reduction at 3T: Comparison of STIR and Slice Encoding for Metal Artifact Correction Fat-suppressed T_2_-weighted Images

**DOI:** 10.2463/mrms.mp.2015-0055

**Published:** 2016-02-20

**Authors:** Young Han LEE, Seok HAHN, Eunju KIM, Jin-Suck SUH

**Affiliations:** 1 Department of Radiology, Research Institute of Radiological Science, Medical Convergence Research Institute, and Severance Biomedical Science Institute, Yonsei University College of Medicine, 50-1 Yonsei-ro, Seodaemun-gu, Seoul 03722, Republic of Korea; 2 Clinical Science, Philips Healthcare

**Keywords:** magnetic resonance, metallic artifact reduction, slice encoding for metal artifact correction, fat suppression, spine

## Abstract

**Purpose:**

To compare short tau inversion recovery (STIR) images with slice encoding for metal artifact correction (SEMAC)-corrected magnetic resonance imaging (MRI) of spectral presaturation with inversion recovery (SPIR) or inversion recovery (IR) at 3T in patients with metallic spinal instrumentation.

**Methods:**

Following institutional review board’s approval, 71 vertebrae with interbody fixation in 26 patients who underwent transpedicular spondylodesis with spinal metallic prostheses were analyzed with SEMAC spinal MRI. All the fixated vertebrae were examined with STIR, and 41 vertebrae of 15 patients were scanned with SEMAC-SPIR T_2_-weighted MRI. The remaining 30 vertebrae of 11 patients were scanned with SEMAC-IR T_2_-weighted MRI. Two musculoskeletal radiologists compared the image sets and qualitatively analyzed the images with a five-point scale that included artifact reduction around the metallic implant and visualization of the rod and pedicle. Quantitative assessments were performed by calculating the signal intensity ratio of the fixated vertebra and non-metallic vertebra and by calculating the signal-to-noise ratios (SNRs) of the vertebrae. A paired *t*-test was used for the statistical analyses.

**Results:**

The SEMAC-IR MRI had a significant decrease in the metallic artifact area (*P* < 0.05), while the SEMAC-SPIR MRI yielded significantly increased artifact areas (*P* < 0.05). However, the signal intensity ratios (i.e., metal-induced signal pile-up) were not significantly different (*P* > 0.05) between the STIR and SEMAC MRI. The SNR of the SEMAC MRI was significantly lower than the SNR of the STIR (*P* < 0.05). The metal artifact reduction scores were significantly higher in the SEMAC-SPIR MRI (*P* < 0.05).

**Conclusion:**

Despite the relatively larger artifact size and lower SNRs, the SEMAC-SPIR MRI was superior to the other types of fat-suppressed MRI of SEMAC-IR or T_2_-weighted STIR MRI. However, the drawbacks of high signal pile-up, large artifact size, and relatively low SNRs require further investigation to determine the best method for fat-suppressed MRI of metallic implants.

## Introduction

Post-operative radiologic evaluation of metallic prosthesis-related complications, including peri-prosthetic bone resorption or osteolysis, prosthetic metallic loosening or instability, and prosthesis-related infections have become more prevalent as the frequency of spinal fusion surgery has increased.^1,2^ In the presence of metallic devices, magnetic resonance imaging (MRI) suffers from signal loss, signal pile-up, and image distortion caused by metallic MR artifacts.^3^ Conventional principles of metallic MR artifact reduction techniques include magnetic field strength, pulse sequence type, voxel size, echo train length, and bandwidth.^4,5^ Advanced metal artifact-reducing MR techniques have proven useful in overcoming these metallic artifacts, including slice encoding for metal artifact correction (SEMAC), multi-acquisition variable-resonance image combination (MAVRIC), a combination of SEMAC and MAVRIC,^6–13^ and iterative decomposition of water and fat with echo asymmetry and least-squares estimation (IDEAL).^14^

Fat-suppressed MR images can provide higher tissue contrast by suppressing bright adipose signals, which aids in the detection of small fluid signals.^15–18^ Further, fat suppression on MR images would be beneficial for patients with metallic implants. Several options are available for fluid-sensitive MR imaging in patients with metallic devices including short tau inversion recovery (STIR), spectral presaturation with inversion recovery (SPIR) T_2_-weighted SEMAC MR images, and inversion recovery (IR) T_2_-weighted SEMAC MR images. Recently, reports have indicated that SEMAC-SPIR spinal T_2_-weighted MR images were a feasible approach for reducing metallic artifacts.^18^ Because of the nature of IR, STIR MR imaging has strength for the metallic MR images, and STIR MR imaging is one of the metal artifact reduction solutions in terms of local field inhomogeneity affects.^4,5^

We hypothesize that the application of an advanced metal artifact reducing technique with fat suppression might be useful in metallic MR imaging at 3T MR imaging with increased SNR. Although the feasibility of SEMAC-SPIR images was recently reported,^18^ the advantages of STIR images versus SEMAC-SPIR images and SEMAC-IR images in patients with metallic implant have not yet been determined. As well, to the best of our knowledge, a comparison study of metal artifact reduction in STIR MR images versus SEMAC-SPIR spinal MR images has not been reported. Accordingly, the purpose of the current study was to compare STIR MR images with SEMAC MR images of SPIR or IR on 3T MR imaging in patients with spinal metallic implants.

## Methods

### Study population

Total 53 patients with metallic screws underwent MRI between July 2013 and December 2014. Among 53 patients, 26 patients who underwent STIR MRI and fat-suppressed SEMAC T_2_-weighted spinal MRI using 3T MRI were retrospectively evaluated. The inclusion criteria were as follows: (^1^) patients with posterior spinal instruments and posterior fixation screws and (^2^) patients who had undergone both STIR sagittal MRI and fat-suppressed SEMAC T_2_-weighted spinal MRI on a 3T MR imager. All patients included underwent SEMAC MRI with either SPIR or IR. All patients had an operative history of interbody screw fixations, and an average of 8.7 years between spinal surgery and MRI. Twelve patients were men and 14 were women. The age range of the 26 patients was 25–81 years (mean age ± standard deviation, 56.2 ± 15.8 years). The average interbody fixation segment was 2.7 levels, as 14 patients had two fixated spinal segments, 6 had three fixated segments, 5 had four fixated segments, and 1 had five fixated segments. The reasons for postoperative MRI included back pain in patients with a history of posterior instrumentation fixation (n = 17), neck pain in a patient with a history of anterior instrumentation fixation (n = 1), posterior instrumentation fixation for a burst fracture (n = 2), evaluation of fever in a patient with a history of posterior instrumentation fixation (n = 1), corpectomy with interbody fixation (giant cell tumor = 1, plasmacytoma = 1), and interbody fixation for metastasis (hepatocellular carcinoma = 1, lung cancer = 1, colon cancer = 1). The study protocol was reviewed and approved by the institutional review board and informed consent for MRI was obtained from all patients.

### MRI protocol

All MR image scans were performed on a 3T MR scanner (Achieva 3.0T TX, Philips Healthcare, Best, The Netherlands) using a dedicated sensitivity encoding (SENSE) spine coil (Philips Healthcare). For fat-suppression images, STIR and SEMAC T_2_WI with SPIR or with IR images were compared.. The sagittal T_2_-weighted STIR MR images were acquired as follows: repetition time (TR) of 3930 ms, an echo time (TE) of 60 ms, an inversion time (TI) of 210 ms, an echo train length (ETL) of 23, a field of view (FOV) of 260–280 × 260–280 mm, a slice thickness of 4 mm (interslice gap = 0 mm), and an acquisition matrix of 468 × 368 (reconstructed matrix 512 × 512). The average scan time for the T_2_-weighted STIR MR images was approximately 4 min and 19 s. The SEMAC-SPIR sagittal T_2_-weighted MR protocol was as follows: TR of 2000 ms, TE of 120 ms, FOV of 280 × 280 mm, acquisition matrix of 288 × 271 (reconstructed matrix 400 × 400), slice thickness of 4 mm (interslice gap = 0 mm), and SEMAC factor of 11. The SEMAC factor is the number of additional Z (through-plane) phase encoding steps. The average scan time for the SEMAC-SPIR MR images was approximately 4 min and 50 s. The SEMAC-IR sagittal T_2_-weighted MR protocol was as follows: TR of 3692 ms, TE of 120 ms, TI of 170 ms, FOV of 260–280 × 260–280 mm; acquisition matrix of 288 × 288 (interpolated to 400 × 400), slice thickness of 4 mm (interslice gap = 0 mm), and SEMAC factor of 11. The average scan time of the SEMAC-IR was approximately 6 min and 35 s. SEMAC MR images were reconstructed from the raw MR images on the MR console after MR scanning, and the average reconstruction time of the SEMAC images was approximately 2 min.

### Image analyses

The STIR MR images obtained of 71 vertebrae of 26 patients were compared to SEMAC-SPIR MR images (n = 30 vertebrae of 11 patients) and SEMAC-IR MR images (n = 41 vertebrae of 15 patients). All image sets were assessed by means of consensus by two musculoskeletal fellowship-trained radiologists with expertise in spine MR imaging (Y.H.L. and S.H. with 10 and 2 years of experience in spinal MRI, respectively). Five continuous sagittal images in the center of the pedicle screw were selected for qualitative and quantitative image analyses for each vertebra. Image analysis was performed using commercially available PACS Centricity® Radiology software (RA1000, GE Healthcare, Barrington, Illinois, USA).

The image quality was evaluated in terms of metallic implant/pedicle visualization and artifact reduction around the rod/pedicle. The qualitative analysis was performed using a five-point scale for all image sets as follows: (^1^) visualization of the rod/pedicle (grade 1, nearly complete lack of prosthesis visualization; grade 2, visualization of less than one third; grade 3, visualization of one-third to two-thirds; grade 4, visualization of more than two-thirds; and grade 5, delineation of the entire prosthesis with clear screw pitch) and (^2^) artifact reduction around the metallic implant/ prosthesis (grade 1, artifacts obscured in the whole vertebra; grade 2, artifact obscured more than half of the vertebra including pedicle; grade 3, artifact obscured less than half of the vertebra; grade 4, artifact within 0.5 cm; grade 5, presence of artifact but clear delineation of bone-prosthesis interface).

**Fig. 1.**
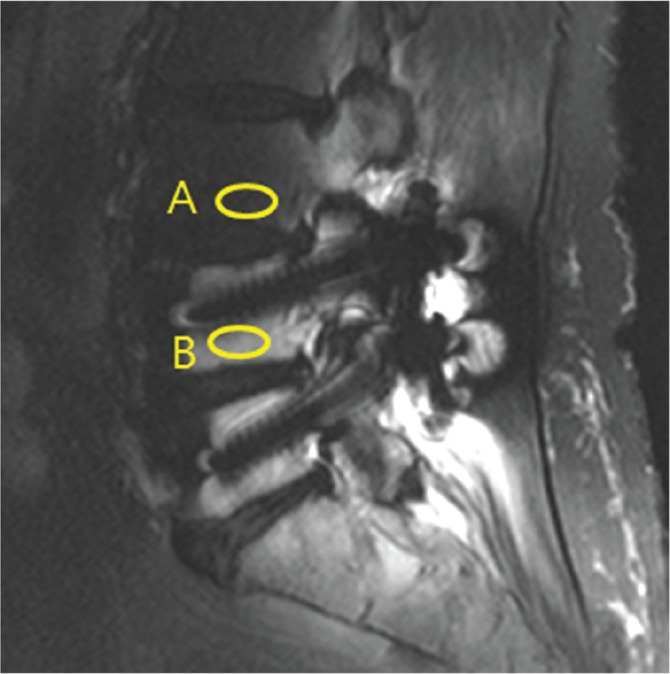
Magnetic resonance (MR) image indicating the measurements of marrow signal intensity in an oval shape region-of-interest (ROI) approximately 60 mm^2^ size encompassing the screw-fixated L4 bone marrow (ROI, **B**) and L3 bone marrow (ROI, **A**).

Quantitative assessments were performed using approximately 60 mm^2^ region-of-interest (ROI) drawings in each vertebra from all image sets (Fig. 1). For evaluation of the signal intensity of the bone marrow around the metallic implant, the ROI means were recorded. The measurements of the ROIs were repeated with a 2-day interval between repeated analyses, and the mean values of the ROIs were used. The ratio of the signal intensity from the free-hand drawn artifact and the fixated vertebra and the signal intensity of the adjacent vertebra was calculated. To measure the area of high signal pile-up, the high signal intensity regions around the metallic implant were measured using free-hand drawings for comparison of high signal pile-up. The standard deviations of the background were measured for calculation of the signal-to-noise ratio (SNR). The SNR was calculated as the ratio of the mean signal intensity of non-fixated vertebra to the standard deviation of the signal in an ROI placed in the background.

For statistical analyses, the ratio, areas, SNRs, and scores were compared between the MR image sets with paired *t*-tests. All statistical analyses were performed using statistical software (*R* package 3.1.1; http://cran.r-project.org). *P* values <0.05 were considered statistically significant.

## Results

In comparison among STIR, SEMAC-SPIR, and SEMAC-IR MR images, the signal intensity ratios were higher in the SEMAC-SPIR MR images and lower with the IR sequence of STIR and SEMAC-IR (*P* > 0.05). The areas were higher with the SEMAC-SPIR and lower with the IR sequence of STIR and SEMAC-IR (*P* < 0.05). Both SEMAC MR images with FS and IR exhibited lower SNRs than conventional STIR images (Table 1).

**Fig. 2.**
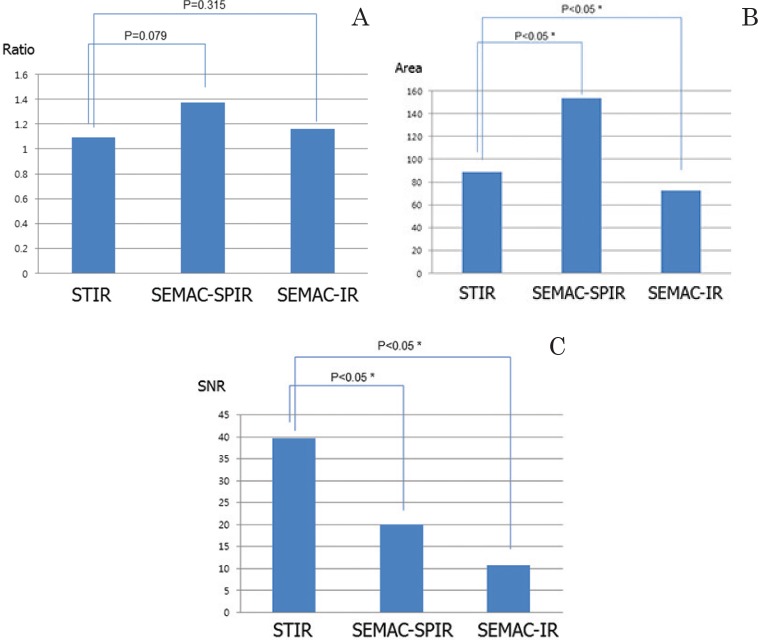
The graph shows a comparison between STIR, SEMAC-SPIR, and SEMAC-IR MR images. (**A**) The signal intensity ratios of screw-fixated vertebra and adjacent vertebra. In general, the signal intensity ratios were higher in the SEMAC-SPIR MR images and lower with the IR sequence of STIR and SEMAC-IR. However, the differences were not statistically significant. (**B**) The areas of high signal intensity. Like former signal intensity ratios, the areas were higher with the SEMAC-SPIR and lower with the IR sequence of STIR and SEMAC-IR. Further, the differences were statistically significant. (**C**) The SNRs. Both SEMAC MR images with SPIR and IR exhibited lower SNRs than conventional STIR images. IR, inversion recovery; MR: magnetic resonance; SEMAC, slice encoding for metal artifact correction; SNR, signal-to noise ratio; SPIR, spectral presaturation with inversion recovery; STIR, short tau inversion recovery.

SEMAC-IR MR images enabled a significant decrease in the metallic artifact areas (*P* < 0.05), while SEMAC-SPIR MR images yielded significantly increased artifact areas (Fig. 2B). However, the signal intensity ratios (i.e., metal-induced signal pile-up) of SEMAC-IR and SEMAC-SPIR MR images were not significantly different (*P* > 0.05; Fig. 2A). In addition, the SNRs of SEMAC-MR images were significantly lower than those of STIR MR images (*P* < 0.05; Fig. 2C).

**Fig. 3.**
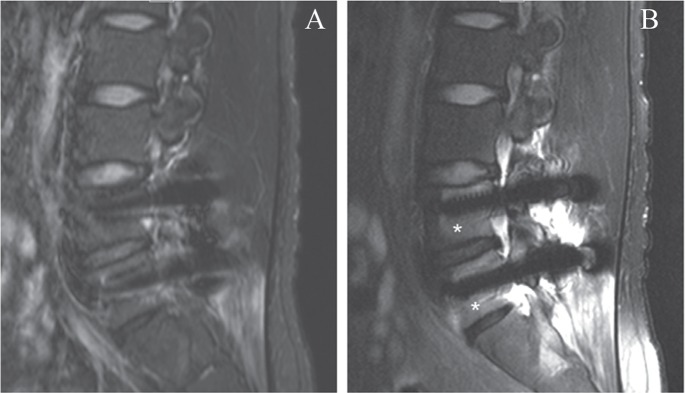
A 32-year-old man with a history of posterior lumbar interbody fixation of L4-5. The STIR sagittal MR image (**A**) and SEMAC-SPIR MR image (**B**) showed metallic susceptibility artifacts around the metallic prostheses. The SEMAC-SPIR MR image showed a more severe signal pile-up (asterisks). The pedicle prostheses were more clearly visualized on SEMAC-SPIR images (B), but the STIR MR image was generally more homogeneous (A). IR, inversion recovery; MR: magnetic resonance; SEMAC, slice encoding for metal artifact correction; SPIR, spectral presaturation with inversion recovery; STIR, short tau inversion recovery.

**Fig. 4.**
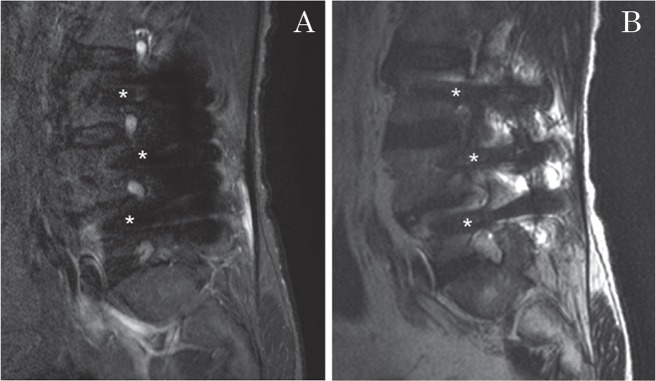
A 55-year-old man with a history of posterior lumbar interbody fixation of L3–4–5. (**A**) The STIR sagittal image showed more metallic susceptibility artifacts around the metallic prostheses (asterisks). (**B**) The SEMAC-IR MR image at the same level showed less metallic artifacts. However, the SNR of SEMAC-IR was lower than that of STIR. IR, inversion recovery; MR: magnetic resonance; SEMAC, slice encoding for metal artifact correction; SPIR, spectral presaturation with inversion recovery; STIR, short tau inversion recovery.

**Fig. 5.**
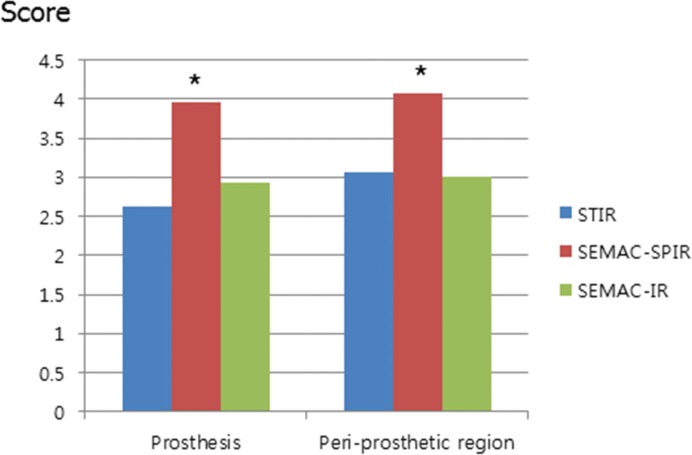
The graph shows a comparison of the metallic artifact reduction scorings. The scores of metal artifact reduction of the visualization of prosthesis and peri-prosthetic region were higher in SEMAC-SPIR. Asterisks indicate statistically significant. SEMAC, slice encoding for metal artifact correction; SPIR, spectral presaturation with inversion recovery.

Although the signal pile-up was more severe in SEMAC-SPIR MR images than in STIR MR images, the delineation of the rod-bone interface was relatively good in the SEMAC-SPIR MR images (Fig. 3). The signal pile-up was improved in SEMAC-IR MR images; however, the corresponding SNR was low (Fig. 4). Further, the metal artifact reduction scores of the metallic prosthesis (4.07 of SEMAC-SPIR vs. 3.13 of SEMAC-IR and 3.07 of STIR) and peri-prosthetic region (3.95 of SEMAC-SPIR vs. 2.93 of SEMAC-IR and 2.63 of STIR) were significantly higher in SEMAC-SPIR MR images (Table 2, Fig. 5).

## Discussion

Bone marrow abnormalities tend to have long T_1_ and T_2_ values, which result in higher signal intensities than values obtained from muscles. Because detection of pathologic tissue is enhanced using fat-saturation MR imaging, fat suppression MR images are commonly utilized in spine MR imaging to suppress the signal from adipose tissue, and enhance the detection of abnormal tissue. The STIR MR images are superior to the T_2_-weighted MR images for depicting bone marrow abnormalities with comparable scan times and image quality.^15,16,19,20^ In patients with metallic devices, the fat-suppressed MR image could be useful for detection of metallic prosthesis-related pathology; however, in metallic MR imaging fat saturation often yields poor quality MR images with severe distortion and artifacts. Because the 3T MR imager offers the advantage of increased SNR and high signal intensity, fat-suppressed SEMAC using 3T MR imaging would be a major improvement. Recently, the feasibility of SEMAC-SPIR MR images using 3T MR imaging was introduced;^18^ however, the evaluations conducted did not include a comparison of STIR MR images and SEMAC-IR MR images. For this reason, we assessed fat-suppressed SEMAC MR images with SPIR and IR, and compared these SEMAC MR images with STIR MR images in patients with metallic devices.

Established drawbacks of SEMAC images include relatively long scan times, which results in a low matrix,^6,11,18^ and a decreased SNR. The size of the metallic artifact was lower on SEMAC-IR MR images; however, the SNRs of SEMAC-IR MR images were low. Further, the SNRs of the SEMAC-SPIR and SEMAC-IR MR images were significantly lower than the STIR MR images, with the SNRs of the SEMAC-IR being the lowest. Nonetheless, the fat-suppressed SEMAC-SPIR corrected T_2_-weighted MR images, as well as STIR, did not overcome high signal pile-up. However, the overall scorings were higher in SEMAC-SPIR MR images despite the relatively larger artifact size and lower SNRs (Fig. 3). However, this paradoxical phenomenon could be a result of the inhomogeneous and incomplete fat-saturation of frequency-selective SEMAC-SPIR MR images (Fig. 5), which was previously reported.^18^

Metallic MR artifacts resulted from the sum of local field inhomogeneities due to spin resonance differences between the metal and surrounding soft-tissue, which altered both frequency and phase.^4^ The metallic MR artifact might be exacerbated in fluid-sensitive fat-suppressed MR images. Though the feasibility of metal artifact-reducing MR techniques, including the fat-suppressed SEMAC-SPIR MR images, has been proven,^18^ the question of whether or not postoperative spine MR images should be acquired with STIR, SEMAC-SPIR, or SEMAC-IR in patients with a metallic device remains unanswered. The results of the current study indicated that the SEMAC-SPIR was superior to SEMAC-IR or STIR of fat-suppressed MR images despite high signal pile-up, large artifact size, and relatively low SNRs.

**Fig. 6.**
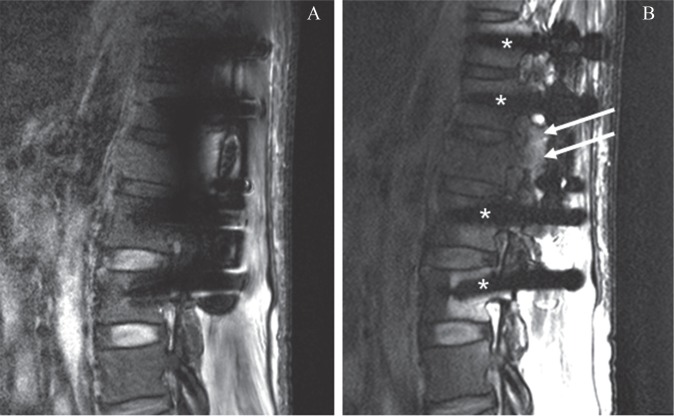
A 57-year-old man with a history of hepatocellular carcinoma (HCC) and posterior fixation of T11-12-L2-3. (**A**) The short tau inversion recovery (STIR) sagittal image showed more metallic susceptibility artifacts around the suspected metastatic lesion of the left pedicle and lamina. (**B**) The SEMAC-SPIR MR image at the same level visualized less metallic artifacts (asterisks) and showed the metastatic lesion (arrows) more definitely, and it was not clearly seen on the STIR image. MR: magnetic resonance; SEMAC, slice encoding for metal artifact correction; SPIR, spectral presaturation with inversion recovery.

SEMAC-SPIR MR images resulted in less in-plane and through-plane artifacts, which improved the delineation of the rod-bone interface despite the high signal pile-up. The SEMAC-SPIR MR imaging was useful with a 3T MR imager. The SEMAC-SPIR MR images showed good overall performance for metallic artifact reduction in prosthesis and peri-prosthetic regions as well as in abnormal lesions (Fig. 6). Furthermore, a hybrid image with SEMAC and MAVRIC should be evaluated in fluid-sensitive MR imaging in patients with metallic devices.

The current study had some limitations. First, details on the metallic component of the metallic prosthetic implants could not be collected because of the unavailability of some surgical notes. Nevertheless, we demonstrated a comparison of STIR and fat-suppressed SEMAC MR images using 3T MR imaging. Second, we compared STIR images with SEMAC-SPIR or SEMAC-IR. Because the scan time was limited, the images compared in the three image sets were not from the same patients. Finally, we evaluated the comparison of STIR and fat-suppressed SEMAC MR images. However, we did not evaluate other advanced MR techniques including MAVRIC, mDIxon (modified Dixon), or IDEAL. Considering the trend of MR metallic artifact reduction, the hybrid techniques should be evaluated in the future to facilitate the use of fat-suppressed MR images in patients with metallic prostheses.

Considering the indication that the number of prosthetic surgeries are increasing,^1,2^ the importance of MR imaging of metallic prosthesis will likely intensify. Fluid sensitive MR imaging is important in postoperative MR evaluation and one of the most widely used and conventional fluid sensitive MR techniques is STIR MR imaging. We compared conventional STIR MR images with advanced SEMAC MR images and determined that the SEMAC-SPIR MR images were superior to the other types of SEMAC-IR or STIR of fat-suppressed MR images. However, the drawbacks of high signal pile-up, large artifact size, and relatively low SNRs should be investigated to determine the best method for fat-suppressed MR images of metallic implants.

## Figures and Tables

**Table 1.** Signal intensity ratios, areas of artifacts, and SNRs of STIR, SEMAC-SPIR, and SEMAC-IR MR images

	STIR	SEMAC-SPIR	SEMAC-IR
Signal intensity ratio	1.09	1.376	1.16
Area of artifacts	88.48	153.30	72.71
SNR	39.73	20.02	10.70

IR, inversion recovery; SEMAC, slice encoding for metal artifact correction; SNR, signal-to-noise ratio; SPIR, spectral presaturation with inversion recovery; STIR, short tau inversion recovery.

**Table 2.** Metal artifact reduction scores of the metallic prosthesis and peri-prosthetic region in STIR, SEMAC-SPIR, and SEMAC-IR MR images

		STIR	SEMAC-SPIR	SEMAC-IR
Score	Prosthesis	2.63	3.95	2.93
	Peri-prosthetic region	3.07	4.07	3.13

IR, inversion recovery; SEMAC, slice encoding for metal artifact correction; SNR, signal-to noise ratio; SPIR, spectral presaturation with inversion recovery; STIR, short tau inversion recovery.
